# Versican enrichment predicts poor prognosis and response to adjuvant therapy and immunotherapy in gastric cancer

**DOI:** 10.3389/fimmu.2022.960570

**Published:** 2022-09-20

**Authors:** Junquan Song, Rongyuan Wei, Shiying Huo, Chenchen Liu, Xiaowen Liu

**Affiliations:** ^1^ Department of Gastric Surgery, Fudan University Shanghai Cancer Center, Shanghai, China; ^2^ Department of Oncology, Shanghai Medical College of Fudan University, Shanghai, China

**Keywords:** VCAN, gastric cancer, cancer associated fibroblasts, adjuvant chemotherapy, immunotherapy

## Abstract

**Background:**

Increasing evidence has revealed an important role of versican (VCAN) on various aspects of cancer progression. Here, we assessed the impact of VCAN expression on prognosis and the response to adjuvant therapy and immunotherapy in patients with gastric cancer (GC).

**Methods:**

Four independent cohorts containing 1353 patients with GC, were utilized to investigate the effect of VCAN expression on prognosis and response to adjuvant therapy in GC. Two cohorts treated with immune checkpoint blockades were included to assess the predict value of VCAN expression on response to immunotherapy. Moreover, the bulk RNA-seq and single-cell RNA-seq data were analyzed to illustrate the role of VCAN in tumor microenvironment. Clinical outcomes of patient subgroups were compared by Kaplan-Meier curves with the log-rank test.

**Result:**

High VCAN expression was associated with poor prognosis for patients with GC. Compared with patients with high VCAN expression, patients with low VCAN expression benefited more from adjuvant chemotherapy and adjuvant chemoradiotherapy. Moreover, patients with high VCAN expression tended to be resistant to immunotherapy, and VCAN could serve as a promising indicator for predicting the response to immunotherapy. VCAN^high^ tumors showed a specific microenvironment with more cancer associated fibroblasts infiltration and significant enrichment of stromal relevant signaling pathways.

**Conclusion:**

VCAN could predict the response to adjuvant chemotherapy, adjuvant chemoradiotherapy and immunotherapy in GC, and designing new medicine target to VCAN might be an effective way to improve the efficacy of several treatment options for GC.

## Introduction

Gastric cancer is one of the most common malignant carcinomas and ranks the fourth leading cause of cancer related death (1). Despite that huge advances in treatment has been achieved in aspects of diagnosis and therapy, patients with GC still have unsatisfactory prognosis (2, 3). Surgery and postoperative adjuvant therapy are the main treatments for GC, and immunotherapy has become an increasingly important part of treatment in the past few years and demonstrated the powerful effect of regressing tumors (4, 5). However, a large number of patients do not respond to these therapies, and it is urgent to explore therapy resistance mechanisms and seek effective biomarkers to better guide clinical treatment.

VCAN, an extracellular matrix proteoglycan, plays an important role in many aspects of organ development and disease (6, 7). VCAN interacts with diverse extracellular matrix (ECM) components like tumor necrosis factor-stimulated gene-6, CD44 and toll-like receptors, all of which are crucial in tissue inflammation caused by infection and injury (8). Increasing studies have shown VCAN is involved in various aspects of cancer progression, including cell proliferation, metastasis, and angiogenesis (9). Moreover, VCAN has been reported to be enriched in chemotherapy-resistant patients with cervical cancer (10). Versican silencing improved the antitumor efficacy of endostatin by alleviating its induced accumulation of myeloid-derived suppressor cells (MDSCs), tumor-associated macrophages (TAMs) and inflammatory cytokines in the tumor microenvironment (11). However, the impact of VCAN on response to adjuvant therapy and immunotherapy response remains unclear in GC.

In this study, multiple independent cohorts were used to explore the relationship between VCAN expression and response to adjuvant therapy and immunotherapy in GC. Single cell RNA sequencing and bulk RNA sequencing data was utilized to explore the role of VCAN in tumor microenvironment. Through the analysis of multiple omics and independent cohorts, we comprehensively explored the negative effect of VCAN on anti-tumor therapeutic efficacy and its potential mechanisms. We found that patients with low VCAN expression benefited more from adjuvant chemotherapy, adjuvant chemoradiotherapy and immunotherapy, which was associated with cancer associated fibroblasts. Taken together, this study demonstrated the crucial role of VCAN in response to adjuvant therapy and immunotherapy in GC.

## Methods

### Clinical specimens and follow-up

The tissue microarray of FUSCC cohort, comprising 233 samples with gastric cancer who received gastrectomy without neoadjuvant chemotherapy or radiotherapy between November 2008 and June 2010 at the Department of Gastric Surgery, Fudan University Shanghai Cancer Center (Shanghai, China) was used in this study. All GC tissues were collected after received informed consent from patients. The study protocol was approved by the Clinical Research Ethics Committee of Fudan University Shanghai Cancer Center. All patients experienced follow-up every 6 months until November 2015. Overall survival (OS) was defined as the time from surgery to death or the end of follow-up, and recurrence free survival (RFS) was defined as the time from surgery to recurrence/metastasis or the end of follow-up.

### Immunofluorescence staining

The automatic immunohistochemical staining machine (Leica, Bond III, Germany) was used for dewaxing and antigen retrieval. After five rinses with phosphate-buffered saline (PBS), tissue array was soaked in hydrogen peroxide solution, incubated at room temperature for 10 min. Then, VCAN antibody (Abcam, ab177480, USA, 1:600) was added to the tissue array, incubated at 37°C for 1 h. After five rinses with PBS, goat anti-rabbit poly-HRP (Leica, DS9800, Germany) was added to the tissue array, incubated at 37°C for 10 min. Dewaxing and antigen repair were performed again. FAP antibody (Abcam, ab207178, USA, 1:250) was added to the tissue array, incubated at 37°C for 1 h. After five rinses with PBS, goat anti-rabbit poly-HRP (Leica, DS9800, Germany) was added to the tissue array, incubated at 37°C for 10 min. The nucleus was stained with DAPI. Finally, 3DHISTECH fluorescence imaging scanner was used for scanning, and HALO platform was used for quantitative analysis of staining results. The percentage of VCAN positive cells was used to identify the expression level of VCAN protein.

### Data sources

Gene expression profiles in the form of fragments per kilobase million (FPKM) and corresponding clinical information of 33 human cancers in the Cancer Genome Atlas (TCGA) were collected from the UCSC XENA (https://xenabrowser.net/datapages/) website. The FPKM values were transformed to transcripts per kilobase millions (TPM) values. Specific information about 33 cancer types could be found in **Supplementary Table 1**. The gene expression profiles and corresponding clinical information of Asian Cancer Research Group (ACRG) cohort (GSE66229), SMC cohort (GSE26253) and MD Anderson Cancer Center (MDACC) cohort (GSE28541) were gathered in this study for further analysis, which were acquired from the Gene Expression Omnibus (GEO) database (https://www.ncbi.nlm.nih.gov/geo/). In SMC cohort, all patients received curative gastrectomy and INT-0116 regimen (5-fluoouracil/leucovorin and radiation) as adjuvant treatment (12). All patients of the MDACC cohort underwent neoadjuvant chemotherapy or chemoradiation therapy (13). For the microarray data from Affymetrix^®^, we got raw “CEL” file from GEO database and adopted the robust multiarray averaging method with the “affy” and “simpleaffy” packages to standardize the microarray data. For the microarray data from other platforms, we downloaded directly the normalized matrix files. We obtained the PD-L1 treatment cohorts for gastric cancer (KIM cohort) and melanoma (Hugo cohort) from Tumor Immune Dysfunction and Exclusion (TIDE) database (http://tide.dfci.harvard.edu). Processed gastric cancer single-cell gene expression data (GSE167297) was composed of deep layer (D1, D2, D3, D4, D5) and superficial layer (S1, S2, S3, S4, S5) of tumor tissues and paired normal tissues (N1, N2, N3, N4), which were downloaded from GEO database.

### Single-cell RNA sequencing data analysis

The single-cell gene expression data was analyzed by the R package “Seurat”. Firstly, we eliminated low-quality cells on the basis of the number of genes, RNA and the proportion of mitochondrial genes in each cell. All samples including the rest of cells were integrated into a single profile and batch-effect was adjusted with R package “Harmony”. Then, Principal component analysis (PCA) was performed on 1500 genes with significantly different levels of expression after log-normalization and homogenization. Moreover, Uniform manifold approximation and projection (UMAP) algorithm was employed to make further dimensionality reduction and marker genes were figured out through “FindALLMarker” function. Finally, the cell lineage for every cluster was annotated according to the marker genes compared to the cell lineage markers in the CellMarker and PanglaoDB database.

### Immune infiltration analysis and gene set enrichment analysis

The immune infiltration among different types of cancers were estimated by multiple algorithms of “IOBR” R package, which integrated a series of existing algorithms for easy comparison and selection (14). Spearman and distance correlation analysis were used to calculate the correlation of VCAN expression and multiple immune cells. The underlying mechanisms of VCAN in the progression of gastric cancer was explored with gene set enrichment analysis (GSEA) method. Firstly, we divided the STAD samples into the high and low group according to the median expression of VCAN in all samples, calculated the differences between the two groups, and arranged differential gene by the value of the foldchange. Then the Hallmarker and Kyoto Encyclopedia of Genes and Genomes (KEGG) gene sets on the basis of prior biological knowledge were used to analyze all samples with GSEA method using the R package clusterProfiler (version 4.0.5). We regarded the normalized enrichment score (NES) and false discovery rate (FDR) as the indicators of enrichment, (Gene sets with |NES|>1 and FDR<0.25 were considered to be possessed with significant enrichment) and utilized the R package enrichplot (version 1.12.1) to visualize the results.

### Statistical analysis

All statistical calculations were performed with R software (version 4.1.1). The Wilcoxon rank sum test was employed to analyze the differences between two groups, while the comparison of differences between three groups or more groups was calculated through the one-way ANOVA or Kruskal–Wallis test. The OS, progressive free survival (PFS) and RFS for patients were estimated by Kaplan-Meier curves, the differences were evaluated by log-rank test, and the cutoff points were selected by “maxstat” R package. The receiver operating characteristic (ROC) curve was implemented to analyze the sensitivity and specificity of immunotherapy response prediction of VCAN expression, and the area under the curve (AUC) was assessed using pROC R package.

## Result

### Landscape of VCAN expression in gastric cancer

Based on “TCGA Pan-Cancer” cohort, we compared the differences of VCAN expression in human pan-cancer and found that VCAN was widely over-expressed in tumor tissues, such as BRCA, CHOL, COAD, ESCA, GBM, HNSC, KIRC, KIRP, LIHC, LUAD, LUSC, STAD, THCA. In addition, the level of VCAN expression diminished in KICH and PCPG (**Supplementary Figure 1A**). To further explore the landscape of VCAN expression in gastric cancer, we assessed the mRNA expression level of STAD samples from the TCGA database (TCGA cohort, n = 388) and GSE66229 dataset (ACRG cohort, n = 300). Compared to normal tissues, the level of VCAN expression significantly increased in tumor tissue in STAD (**Supplementary Figures 1B, F**). Similarly, the level of VCAN expression in tumor tissue was significantly higher than that in surrounding normal tissue from the same sample (**Supplementary Figure 1C**). Moreover, the level of VCAN expression was correlated with pathological stages and significantly up-regulated level of VCAN expression was observed in advanced gastric cancer in comparison to early gastric cancer (**Supplementary Figure 1D**).

Molecular subtypes of gastric cancer were established to facilitate the stratification of patients and the implementation of precision therapy (15). In TCGA cohort, the patients were divided into four subtypes including microsatellite instability (MSI), genome stable (GS), Epstein-Barr virus (EBV) and chromosomal instability (CIN). Compared to the CIN and GS subtypes, the MSI and EBV subtypes possessed the lower VCAN expression (**Supplementary Figure 1E**). The higher VCAN expression was significantly concentrated on epithelial mesenchymal transformation (EMT) subtype in ACRG cohort (**Supplementary Figure 1G**).

### High expression of VCAN was associated with poor prognosis of patients with GC

**Figure 1:**
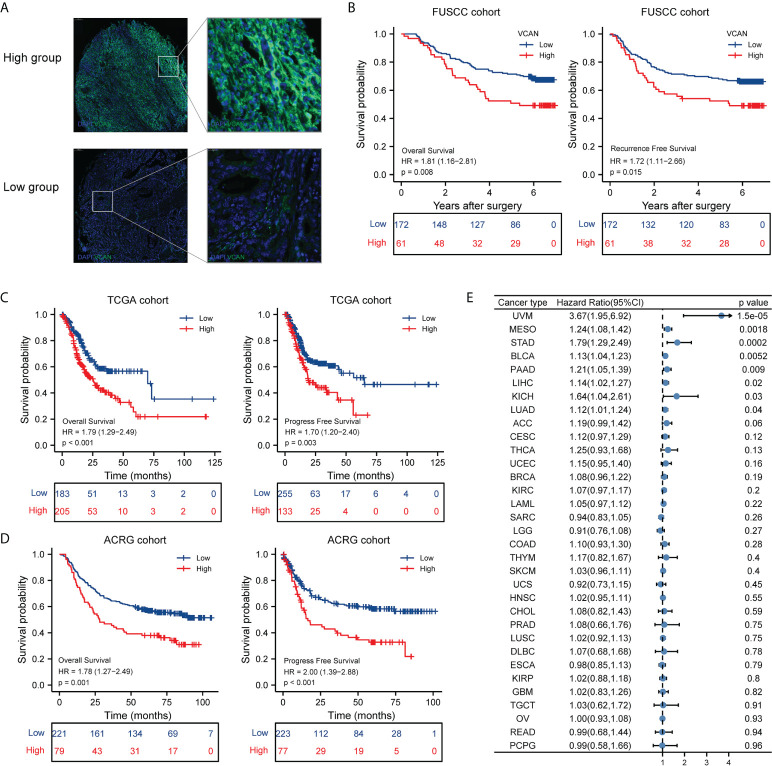
Correlation between VCAN expression and overall survival and progressive free survival. **(A)** Representative immunofluorescence images of the low VCAN group and high VCAN group in FUSCC cohort. **(B)** Kaplan–Meier curves of overall survival (OS) and recurrence free survival (RFS) of low VCAN and high VCAN group in FUSCC cohort. **(C)** Kaplan–Meier curves of OS and progress free survival (PFS) of low VCAN and high VCAN group stratified in TCGA cohort. **(D)** Kaplan–Meier curves of OS and PFS of low VCAN and high VCAN group stratified in ACRG cohort. **(E)** The correlation between VCAN expression with OS in human pan-cancer (Pan-cancer Atlas, TCGA).

To further explore the role of VCAN in GC, we constructed tissue microarray of FUSCC cohort containing 233 patients with GC and quantified the expression of VCAN protein using immunofluorescence experiments. Based on the expression of VCAN protein, we divided the patients into the high group (The proportion of VCAN positive cells was more than 19%) and low group (The proportion of VCAN positive cells was less than 19%), and the representative immunofluorescence images of the high group and low group was shown in **Figure 1A**. The association between VCAN expression and clinicopathologic features in FUSCC cohort was showed in **Supplementary Table 2**. Survival analysis indicated that high level of VCAN expression was significantly associated with OS and RFS of patients with GC (**Figure 1B**). Similarly, VCAN expression significantly affected OS and PFS of patients in the TCGA cohort (**Figure 1C**) and ACRG cohort (**Figure 1D**). The 5-year survival rate and 5-year progress-free rate of patients in the high VCAN group were significantly lower than those in the low VCAN group (**Figures 1B–D**). Moreover, we also explored the relationship between VCAN expression and clinical outcome in human pan-cancer. The result showed that the OS of VCAN high expression group was poorer than that in the low expression group in multiple cancers (**Figure 1E**).

### VCAN acted as a promising prognosticator for the response to adjuvant therapy in GC

**Figure 2:**
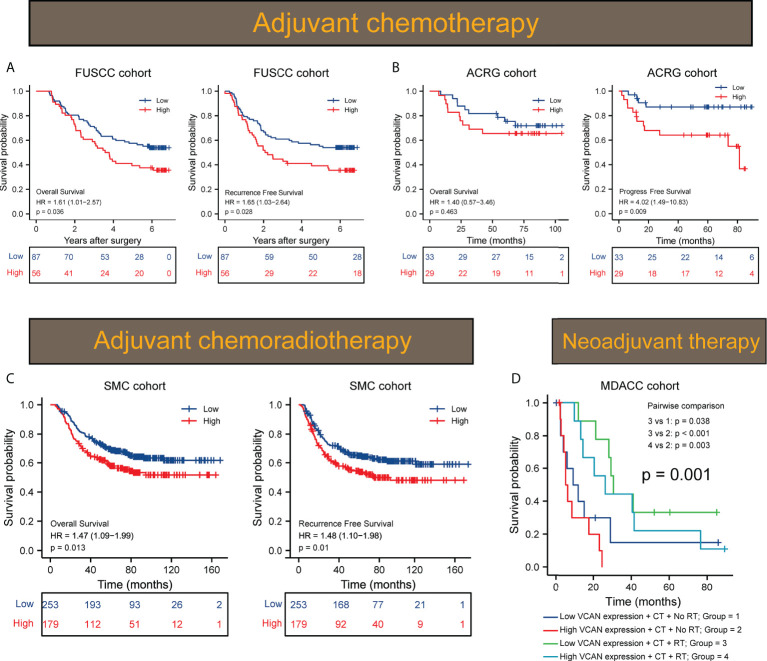
VCAN expression predicted response to adjuvant therapy in GC. **(A)** Kaplan–Meier curves of OS and RFS of low VCAN and high VCAN group in patients treated by ACT (n = 143) in FUSCC cohort. **(B)** Kaplan–Meier curves of OS and PFS of low VCAN and high VCAN group in patients treated by ACT (n = 62) in ACRG cohort. **(C)** Kaplan–Meier curves of OS and RFS of low VCAN and high VCAN group in patients treated by adjuvant chemoradiotherapy (SMC cohort, n = 432). **(D)** Kaplan–Meier curves of OS of low VCAN and high VCAN group in patients treated by neoadjuvant chemotherapy or chemoradiotherapy (MDACC cohort, n = 40). CT, chemotherapy; RT, radiotherapy.

Multiple clinical studies have shown that adjuvant chemotherapy (ACT) could prolong the survival of patients with advanced gastric cancer compared with surgery alone (16, 17). However, extracellular matrix acted as the physical barrier to hinder the penetration of chemotherapy drugs. Given the strong correlation between VCAN and extracellular matrix, we evaluated whether the expression of VCAN affected the efficacy of ACT. Thus, we conducted survival analysis aimed to patients who received ACT in FUSCC cohort and ACRG cohort. We found that the VCAN could affect OS and RFS of patients who received ACT in FUSCC cohort, and patients in the VCAN high group benefited less from ACT compared with patients in the VCAN low group (**Figure 2A**). Besides, we assessed the effect of VCAN on the response to ACT of patients in ACRG cohort. High expression of VCAN was associated with poor PFS of patients received ACT in ACRG cohort (**Figure 2B**). The 5-year survival rate and 5-year progress-free rate of patients received ACT in the high VCAN group were significantly lower than those in the low VCAN group (**Figures 2A, B**). These results showed that it was feasible to predict the response to ACT through detecting the expression of VCAN.

Though recent studies indicated patients with GC could not benefit more from postoperative chemoradiotherapy than chemotherapy, adjuvant chemoradiotherapy was still considered one of the available treatment options for patients who have undergone less than D2 dissection (18). We evaluated the effect of VCAN expression on the efficacy of adjuvant chemoradiotherapy in 432 patients who received homogeneous chemoradiotherapy (5-fluoouracil/leucovorin and radiation) after surgery from SMC cohort. The results showed that the high level of VCAN expression was significantly related to poorer OS and RFS of the patients and the 5-year survival rate and 5-year recurrence-free rate of high VCAN group were significantly lower than those of low VCAN group (**Figure 2C**), suggesting that VCAN expression was an unfavorable factor of adjuvant chemoradiotherapy. Then, we assessed the difference of the response to neoadjuvant therapy between high VCAN group and low VCAN group in MDACC cohort, where all patients received neoadjuvant chemotherapy or chemoradiation therapy. The result showed that both the high VCAN group and low VCAN group benefited more from neoadjuvant chemoradiotherapy than neoadjuvant chemotherapy (**Figure 2D**). However, the effect of VCAN on the efficacy of neoadjuvant chemoradiotherapy was not statistically significant, which needed to be treated with caution due to the limited number of patients. In summary, the expression of VCAN significantly affected the efficacy of adjuvant therapy for GC and targeting to VCAN might be an effective way to improve the efficacy of adjuvant therapy for GC.

### VCAN served as an indicator to predict the efficacy of immunotherapy for GC

**Figure 3:**
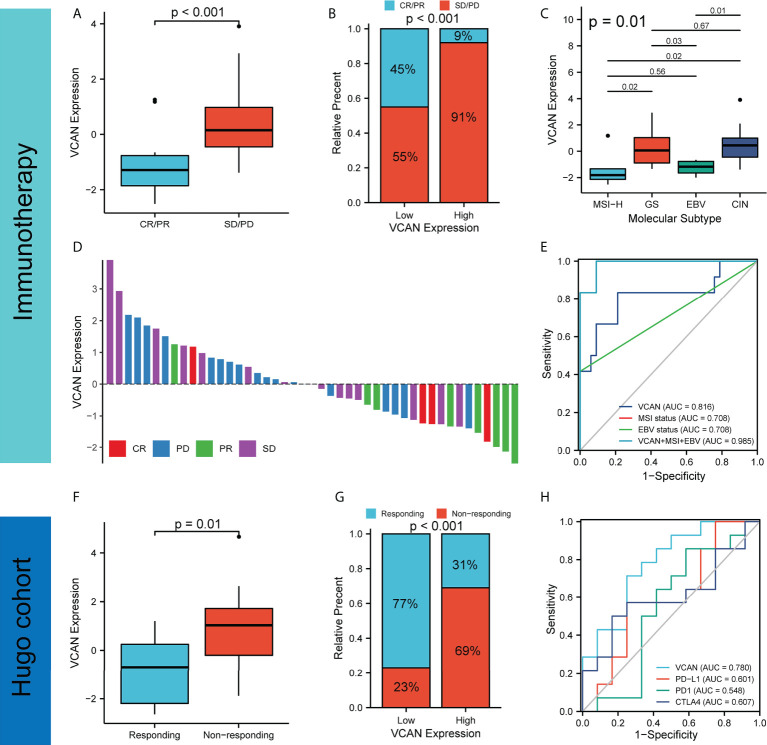
VCAN expression predicted response to immunotherapy. **(A)** The expression of VCAN in complete response (CR)/partial response (PR) group and stable disease (SD)/progressive disease (PD) group in KIM cohort. **(B)** Proportion of CR/PR and SD/PD in low VCAN group and high VCAN group in KIM cohort. **(C)** The expression of VCAN in patients with different molecular subtypes in KIM cohort. **(D)** The expression of in patients with different immunotherapy responses in KIM cohort. **(E)** Receiver operating characteristic (ROC) curves of VCAN expression, MSI status, and EBV status in predicting immunotherapy response in KIM cohort. **(F)** The expression of VCAN in responding group and non-responding group in Hugo cohort. **(G)** Proportion of responding and non-responding to immunotherapy in low VCAN group and high VCAN group in Hugo cohort. **(H)** ROC curves of VCAN, PD-L1, PD1 and CTLA4 expression in predicting immunotherapy response in Hugo cohort.

Immunotherapy that targeted the immune system has revolutionized human cancers treatment, including gastric cancer. Regulation of immune system by immune checkpoint blockade (ICB) led to durable responses in human cancers. Recent clinical trial has shown that nivolumab (the first PD-1 inhibitor) could significantly prolong OS and PFS in patients with advanced gastric, gastro-oesophageal junction, or oesophageal adenocarcinoma (19). Recently, nivolumab (a monoclonal PD-1 antibody) has been approved by the U.S. Food and Drug Administration (FDA) for first-line treatment in patients with advanced or metastatic gastric cancer (20). However, most patients did not respond to immunotherapy, and it was necessary to find specific biomarkers to predict response to immunotherapy for GC (21). We used the KIM cohort (patients with advanced gastric cancer were treated by PD-1 inhibitor) to analyze the relationship between VCAN expression and immunotherapy responses for GC. According to the RECIST 1.1 guidelines, patients in the CR and PR groups were considered responders and patients in the SD and PD groups were considered non-responders (22, 23). We found that the VCAN expression of patients in the stable disease (SD)/progressive disease (PD) group was significantly higher than that in the partial response (PR)/complete response (CR) group (**Figure 3A**), and the proportion of PD/SD in high VCAN expression higher than that in low VCAN expression group, indicating that VCAN expression was not conducive to immunotherapy response (**Figure 3B**). The VCAN expression of patients with different immunotherapy responses was shown in **Figure 3D**. MSI status and EBV status were found to serve as biomarkers for immunotherapy response (20, 24). Interestingly, we found that VCAN expression in patients with MSI-H subtype and EBV subtype was significantly lower than that in patients with GS and CIN subtypes (**Figure 3C**). Then, we constructed the ROC curve to assess the predictive value of VCAN in immunotherapy response. We found that the AUC value of VCAN expression for predicting immunotherapy response was higher than that of MSI status and EBV status, and the AUC value as high as 0.985 when VCAN expression, MSI status and EBV status were combined (**Figure 3E**). Moreover, we also analyzed Hugo cohort (patients with melanoma were treated by PD1 inhibitor) and found patients in non-responding groups had higher VCAN expression than patients in responding group (**Figure 3F**). The proportion of responder for immunotherapy in the high VCAN group was significantly lower than the low VCAN group (**Figure 3G**), and the AUC value of VCAN expression was higher than that of PD-L1, PD1 and CTLA4 expression (**Figure 3H**), which suggested that VCAN might predict immunotherapy efficacy in other cancers. In conclusion, our results showed that patients with high VCAN expression tended to be resistant to immunotherapy, and VCAN could serve as a promising indicator to predict the response to immunotherapy for patients with GC.

### Potential mechanisms by which VCAN affected prognosis and response to therapy for GC

**Figure 4:**
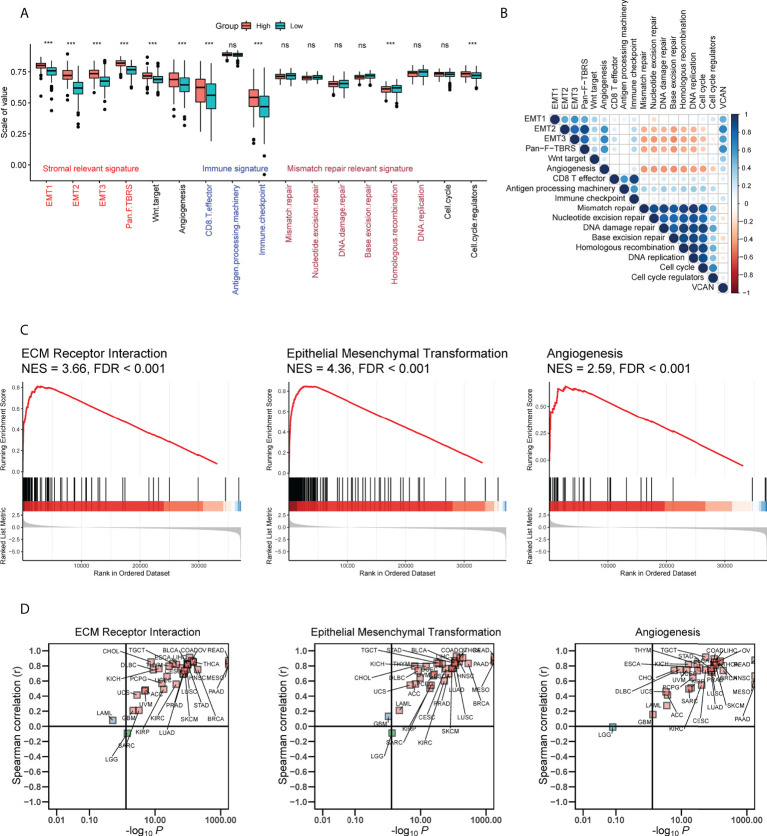
Potential mechanisms by which VCAN affected prognosis and response to anti-tumor therapy in GC. **(A)** The enrichment score of known biological processes in high VCAN group and low VCAN group in TCGA cohort. **(B)** The correlation between VCAN expression and the enrichment score of known biological processes in TCGA cohort. **(C)** Gene set enrichment analysis for patients with high VCAN expression and low expression in TCGA cohort. **(D)** The correlation between VCAN expression and the enrichment score of Extracellular Matrix (ECM) Receptor Interaction signaling, Epithelial Mesenchymal Transformation signaling and Angiogenesis signaling in human pan-cancer (Pan-cancer Atlas, TCGA). ***p < 0.001.

To explore the biological function of VCAN in GC, we quantified the enrichment degree of known biological processes in high VCAN group (the expression of VCAN was higher than the median value) and low VCAN group (the expression of VCAN was lower than the median value) through the “ssGSEA” algorithm in TCGA cohort (25). All of stromal relevant signatures, including EMT1, EMT2, EMT3 and panfibroblast TGFβ response characteristics (Pan-F TBRS), were found to be significantly upregulated in high VCAN group (**Figure 4A**). Correlation analysis confirmed that stromal relevant signatures and angiogenesis were significantly related to VCAN expression (**Figure 4B**). GSEA analysis results showed that VCAN significantly improved ECM Receptor Interaction signaling, Epithelial Mesenchymal Transformation signaling and Angiogenesis signaling (**Figure 4C**). Interestingly, we found that it was in almost human cancers that VCAN expression was positively associated with the enrichment of these signaling pathways (**Figure 4D**), which demonstrated the potential of VCAN as the common target for cancer treatment.

### VCAN expression was positively correlated with cancer associated fibroblasts in the tumor microenvironment

**Figure 5:**
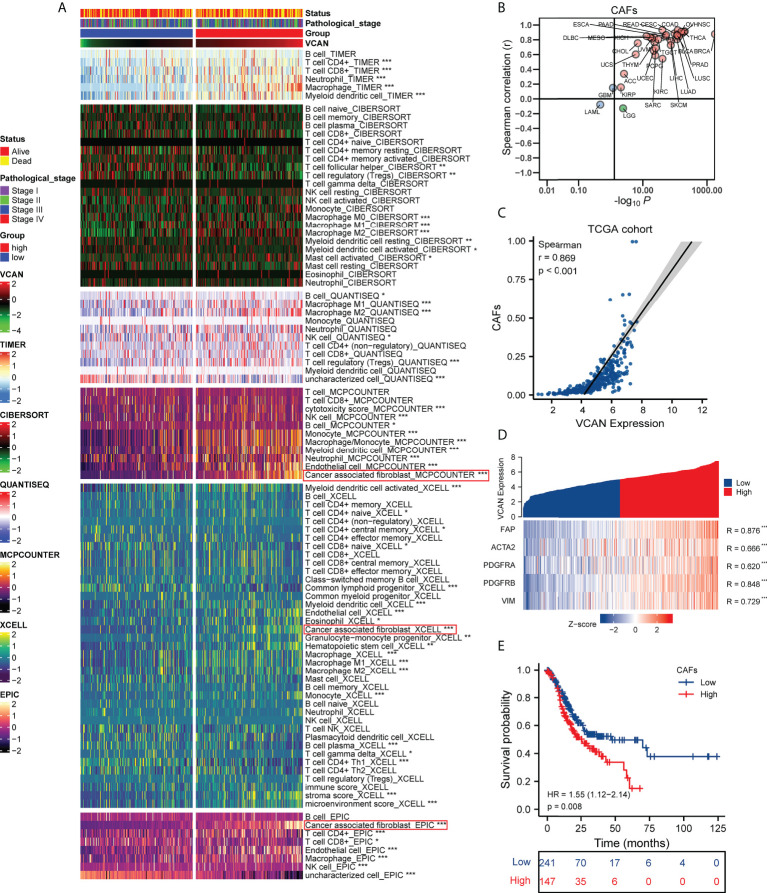
The correlation between VCAN expression and the tumor microenvironment. **(A)** The correlation between VCAN expression with the infiltration of immune cells and stroma cells in tumor microenvironment (TCGA cohort). **(B)** The correlation between VCAN expression with the infiltration of CAFs in human pan-cancer (Pan-cancer Atlas, TCGA). **(C)** The correlation between VCAN expression with the infiltration of CAFs in patients with GC (TCGA cohort). **(D)** The correlation between VCAN expression with the expression of CAFs markers in patients with GC (TCGA cohort). **(E)** Kaplan–Meier curves of OS of low and high group stratified by the CAFs infiltration in patients with GC (TCGA cohort). *p < 0.05, **p < 0.01, and ***p < 0.001. GC: gastric cancer; CAFs: cancer associated fibroblasts.

Tumor microenvironment contained stromal cells and various immune cells, which interacted closely with tumor cells and contributed to tumor progression (26). Multiple algorithms were used to comprehensively assess the landscape of tumor microenvironment in TCGA cohort, including TIMER, CIBERSORT, QUANTISEQ, MCPCOUNTER, XCELL, and EPIC. We found that VCAN was associated with infiltration of a variety of immunosuppressive cells, such as macrophages, T cell regulatory (Tregs), and cancer associated fibroblasts (CAFs) (**Figures 5A, C**). Recent studies revealed that CAFs was the essential cell for depositing and remodeling the extracellular matrix in human cancers (27, 28). VCAN, an extracellular matrix proteoglycan, played an important role in the extracellular matrix remodeling pathway (8). We assumed that VCAN shaping tumor microenvironment was related to CAFs, and conducted the pan-cancer analysis to further analyze the relationship between VCAN and CAFs. The result showed that VCAN was positively associated with the level of CAFs infiltration in the vast majority of human cancers (**Figure 5B**). Fibroblast activation protein (FAP), α-SMA (ACTA2), platelet derived growth factor receptor α/β (PDGFRA/B), and Vimentin (VIM) were widely used as markers to identify CAFs (27). We found that the expression of VCAN mRNA was significantly correlated with the expression of CAFs markers mRNA (**Figure 5D**). To demonstrate the correlation between VCAN and CAFs at the protein level, we conducted multiple immunofluorescence experiments. The result showed that there were the co-existences of VCAN and FAP in the tumor (**Figure S2**), which was consistent with the analysis result at mRNA level. Moreover, survival analysis indicated the infiltration of CAFs was an unfavorable factor for the prognosis of gastric cancer patients (**Figure 5E**). In summary, overexpression of VCAN was often accompanied by the increase in CAFs infiltration in tumor microenvironment, which was detrimental to the prognosis of patients with gastric cancer.

### VCAN was mainly expressed in inflammatory cancer associated fibroblasts

**Figure 6:**
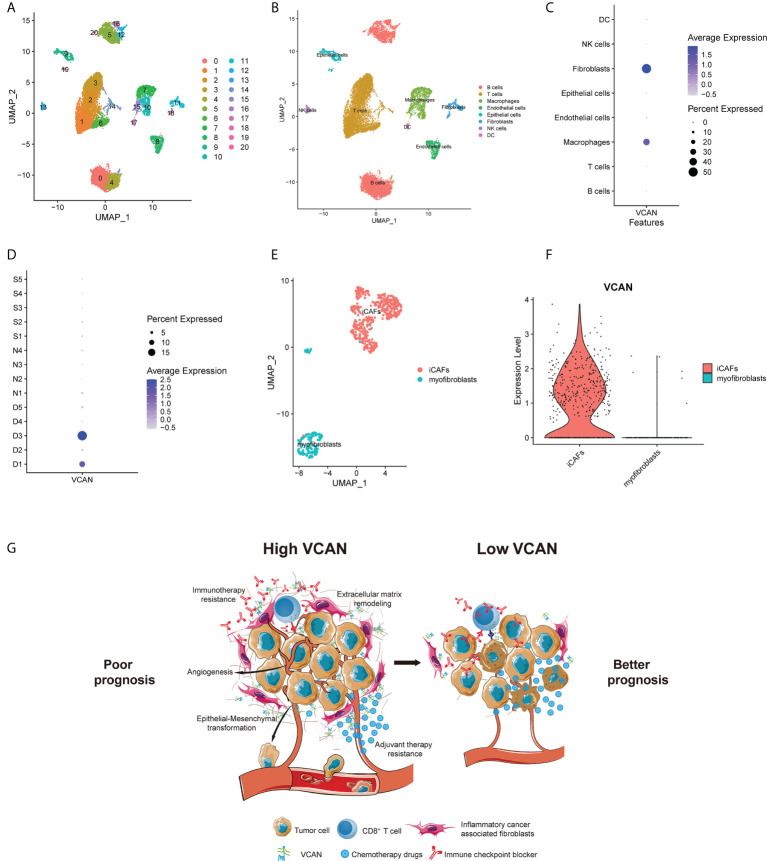
The single cell location of VCAN in the tumor microenvironment. **(A)** UMAP plot showed 21 cell clusters from 19765 cells of patients in GSE167297. **(B)** UMAP plot showed eight cell types from 19765 cells of patients in GSE167297. **(C)** Dotplot showed the expression level of VCAN in different cell types in GSE167297. **(D)** Dotplot showed the expression level of VCAN in deep layer (D1, D2, D3, D4, D5) and superficial layer (S1, S2, S3, S4, S5) of tumor tissues and paired normal tissues (N1, N2, N3, N4) in GSE167297. **(E)** Subpopulation analysis aimed to fibroblasts of patients in GSE167297. **(F)** Violin plot showed the expression of VCAN in iCAFs and myofibroblasts in GSE167297. **(G)** Graphic summary of the proposed model. VCAN secreted by iCAFs was involved in the activation of stroma related pathways, thereby promoting anti-tumor therapy resistance. iCAFs: inflammatory cancer associated fibroblasts.

Single-cell technology could characterize the molecular state of each cell, which enables more in-depth research on the tumor microenvironment and tumor heterogeneity, and it has become an indispensable tool in oncology research (29). To further explore the role of VCAN in tumor environment, gastric cancer single-cell dataset GSE167297 containing deep layer (D1, D2, D3, D4, D5) and superficial layer (S1, S2, S3, S4, S5) of tumor tissues and paired normal tissues (N1, N2, N3, N4) was downloaded and analyzed. After quality control, 19765 cells were eventually included in subsequent analysis and a total of 21 cell clusters were identified through UMAP algorithm (**Figure 6A**). Then, we annotated the cell lineage for every cluster based on the cell lineage marker genes. The single-cell atlas was mainly consisted of immune cells, such as T cells, B cells, Macrophages and dendritic cells (DC). In addition to immune cells, there were non-immune cells (epithelial cells, endothelial cells and fibroblasts) in the single-cell atlas (**Figure 6B**). We found that VCAN was mainly expressed in fibroblasts and macrophages rather than epithelial cells (**Figure 6C**), and the expression of VCAN was concentrated in the deep layers of tumor tissues (**Figure 6D**). Because the bulk RNA-seq analysis revealed the high correlation between VCAN and CAFs infiltration in gastric cancer (**Figure 5C**), we focused on the expression of VCAN in fibroblasts. Fibroblasts were further divided into three subpopulations with unique genetic signatures. Sub-cluster 0 and Sub-cluster 1 were identified as inflammatory CAFs (iCAFs) based on the enriched expression of chemokines such as CXCL1, CXCL14, CCL2, and interleukin 33 (IL33). Sub-cluster 2 had the high expression of ACTA2, therefore it was identified as myofibroblasts (**Figure 6E**). Interestingly, VCAN was mainly expressed in iCAFs and was barely expressed in myofibroblasts (**Figure 6F**). Interestingly, recent study has demonstrated the crucial role of iCAFs in cancer therapy resistance (30). Based on these results, we concluded that VCAN secreted by iCAFs was involved in the activation of stroma related pathways, thereby promoting anti-tumor therapy resistance (**Figure 6G**).

## Discussion

VCAN, known as an extracellular matrix proteoglycan, was mainly constituted by stromal cells (8). Increasing evidence has supported that VCAN overexpression has been implicated in a wide range of malignancies and related to poor prognosis (31). In our study, we found that patients with high VCAN expression displayed worse prognosis in GC. As with our results, it has been documented that VCAN made an impact on the tumor mutation burden and tumor microenvironment of gastric cancer and VCAN lower expression indicated better prognosis and lower grade in GC (32). These reports in combination with our analyses demonstrated the oncogenic part of VCAN in GC. Previous bioinformatic studies also revealed that VCAN was associated with poor prognosis and could serve as a potential independent biomarker for diagnosis and prognosis for patients with GC (33–35). However, whether VCAN expression affected the therapeutic response to anti-tumor treatments remained unclear. Therefore, we mainly focused on the impact of VCAN expression on the efficacy of adjuvant therapy and immunotherapy. Remarkably, this study firstly demonstrated the predictive value of in response to adjuvant chemotherapy, adjuvant chemoradiotherapy and immunotherapy in GC. These results suggested that detection of VCAN expression was conducive to the selection of appropriate treatment and accurate prognostic assessment for patients with GC.

Tumor microenvironment significantly influenced not only tumor progression but also therapeutic response. Tumor microenvironment-mediated therapy resistance resulted from extracellular factors secreted by tumor parenchymal or stromal cells and adhesion of tumor cells to stromal fibroblasts or ingredients of extracellular matrix (36). The development of tumor involved many mechanisms and factors, among which CAFs were regarded as the key components in the tumor microenvironment. CAFs modulated tumor growth, metastasis and therapy responses by remodeling ECM and production of numerous cytokines and chemokines (28). It was reported that the major source of VCAN protein was constituted by CAFs in breast cancer, colon cancer, pharyngeal cancer, ovarian cancer and prostate cancer (31). Similar to previous studies, we found that VCAN was also mainly expressed in CAFs in GC. Upregulation of VCAN in CAFs enhanced ovarian cancer cell motility and invasion potential by activating the NF-κB signaling pathway and upregulated expression of CD44, MMP9 and the hyaluronan mediated motility receptor (37). Specific CAFs clusters could increase PD1 and CTLA4 protein level in Tregs to offer immunotherapy resistance and relevant ECM dysregulation might lead to failure in PD-L1 blockade immunotherapy (38, 39). These studies provided rational explanations for the poor prognosis and the resistance to adjuvant therapy and immunotherapy in the patients with high VCAN expression. Based on the above results, we concluded that targeting VCAN expression in CAFs may be an effective way to inhibit cancer progression and reverse treatment resistance.

Even though we have consolidated and analyzed multiple independent cohorts, multi-centered and randomized clinical trials were still needed to validate our findings. Synthesizing our findings and previous studies, we proposed the mechanism hypothesis of VCAN influencing prognosis and anti-tumor treatment response. However, the mechanism also needs to be validated by *in vivo* and *in vitro* experiments. Meanwhile, we hope that specific inhibitors for VCAN could be developed to improve the effect of anti-tumor therapy in the future studies.

## Conclusions

In conclusion, our findings elucidated that VCAN was correlated with poor prognosis in GC, and patients with high VCAN expression were more prone to resisting adjuvant therapy and immunotherapy. Given the superior prognostic value and predictive value of response to adjuvant therapy and immunotherapy, VCAN could be used as a biomarker and the new therapeutic target for GC.

## Data availability statement

The raw data supporting the conclusions of this article will be made available by the authors, without undue reservation.

## Ethics statement

The studies involving human participants were reviewed and approved by Clinical Research Ethics Committee of Fudan University Shanghai Cancer Center. The patients/participants provided their written informed consent to participate in this study.

## Author contributions

XL and CL for study concept and design, analysis and interpretation of data, drafting of the manuscript, obtained funding and study supervision. JS and RW for acquisition of data, analysis and interpretation of data, statistical analysis and drafting of the manuscript. SH for technical and material support. All authors contributed to the article and approved the submitted version.

## Funding

This study was sponsored Natural Science Foundation of Shanghai (21ZR1414600), Shanghai Pujiang Program (2019PJD007), Shanghai Sailing Program (20YF1409200) and National Natural Science Foundation of China (82002545). The funders had no role in study design, data collection and analysis, decision to publish, or preparation of manuscript.

## Conflict of interest

The authors declare that the research was conducted in the absence of any commercial or financial relationships that could be construed as a potential conflict of interest.

## Publisher’s note

All claims expressed in this article are solely those of the authors and do not necessarily represent those of their affiliated organizations, or those of the publisher, the editors and the reviewers. Any product that may be evaluated in this article, or claim that may be made by its manufacturer, is not guaranteed or endorsed by the publisher.

## Abbreviations

VCAN, Versican; GC, Gastric cancer; CAFs, Cancer associated fibroblasts; OS, Overall survival; RFS, Recurrence free survival; FUSCC, Fudan University Shanghai Cancer Center; SMC, Samsung Medical Center; PBS, Phosphate-buffered saline; ECM, Extracellular matrix; FPKM, Fragments per kilobase million; TCGA, The Cancer Genome Atlas; TPM, Transcripts per kilobase millions; GEO, Gene Expression Omnibus; GSEA, Gene set enrichment analysis; ACRG, Asian Cancer Research Group; MDACC, MD Anderson Cancer Center; TIDE, Tumor Immune Dysfunction and Exclusion; PFS, progressive free survival; IC50, Half maximal inhibitory concentration; ROC, Receiver operating characteristic; AUC, Area under the curve; MSI, Microsatellite instability; GS, Genome stable; EBV, Epstein-Barr virus; CIN, Chromosomal instability; EMT, Epithelial mesenchymal transformation; FAP, Fibroblast activation protein; ACTA2, α-SMA; PDGFRA/B, Platelet derived growth factor receptor α/β; VIM, Vimentin; DC, Dendritic cells; IL33, interleukin 33; ICB, Immune checkpoint blockade; FDA, U.S. Food and Drug Administration; SD, Stable disease; PD, Progressive disease; PR, Partial response; CR, Complete response; Pan-F TBRS, Panfibroblast TGFβ response characteristics.
