# Supplementary material for: Versican enrichment predicts poor prognosis and response to adjuvant therapy and immunotherapy in gastric cancer
Source: Front Immunol. 2022 Sep 20;13:960570. doi: 10.3389/fimmu.2022.960570 (PMC9530562; doi:10.3389/fimmu.2022.960570)
Supplement: Supplementary file 1 [file DataSheet_1.docx]

**Supplementary Materials**

**Figure S1. Landscape of VCAN expression in GC. (A)** The expression of VCAN was compared between tumor tissues and normal tissues in human pan-cancer. **(B)** The expression of VCAN in tumor tissuses and normal tissues of patients with STAD in TCGA cohort. **(C)** The expression of VCAN in tumor tissuse and surrounding normal tissue from the same sample in TCGA cohort. **(D)** The expression of VCAN in patients with different pathological stages in TCGA cohort. **(E)** The expression of VCAN in patients with different molecular subtypes in TCGA cohort. **(F)** The expression of VCAN in tumor tissuses and normal tissues in ACRG cohort. **(G)** The expression of VCAN in patients with different molecular subtypes in ACRG cohort. MSI: Microsatellite instability, GS: Genome stable; EBV: Epstein-Barr virus; CIN: Chromosomal instability; EMT: Epithelial mesenchymal transformation; MSS: Microsatellite stability. *p < 0.05, **p < 0.01, and ***p < 0.001.


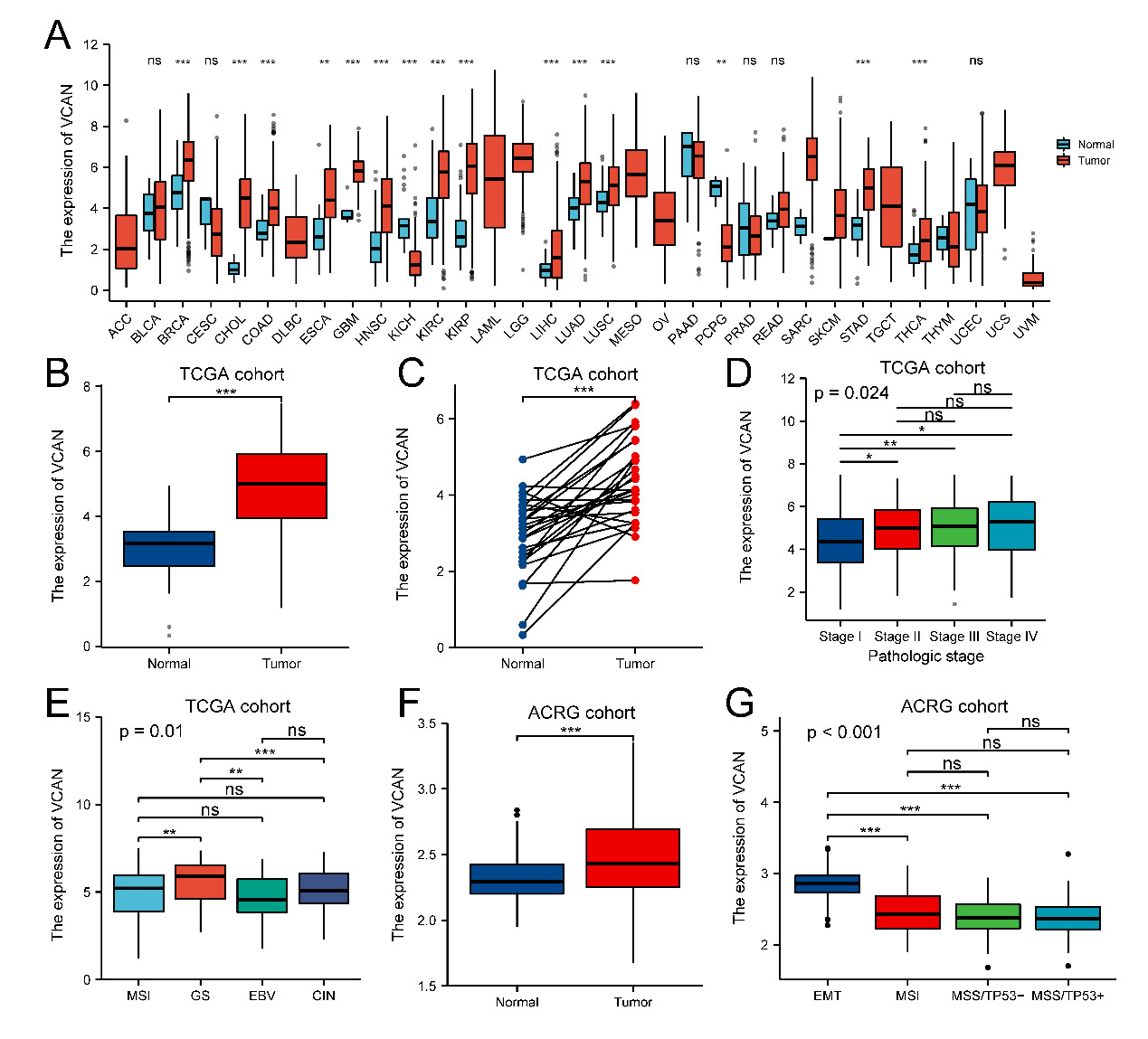


**Figure S2. Representative immunofluorescence images showed the co-existences of VCAN and FAP in FUSCC cohort.** The white arrows refer to VCAN^+^FAP^+^ fibroblasts.

**
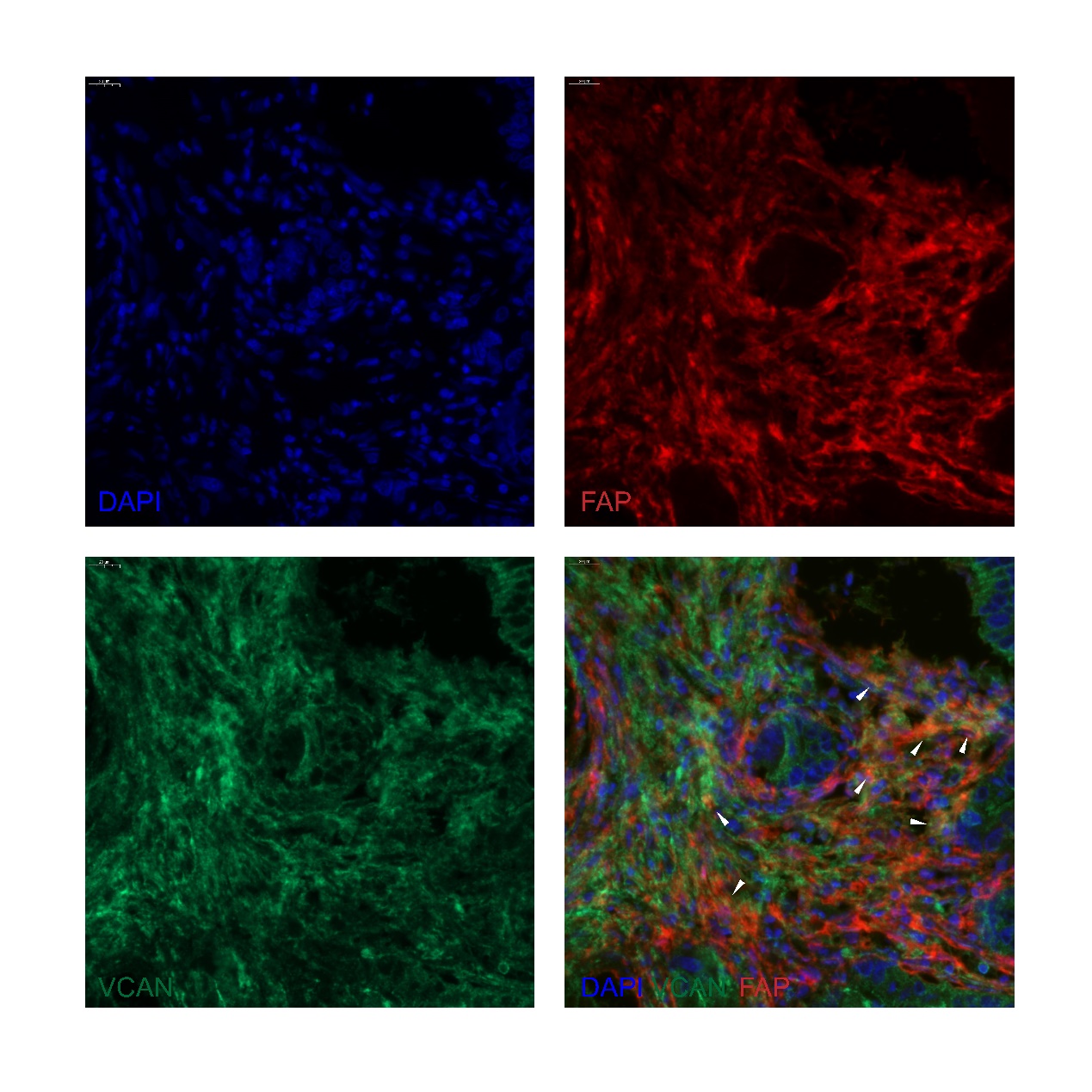
**

**Table S1. Specific information about 33 cancer types.**

| Abbreviation | Full name |
| --- | --- |
| ACC | Adrenocortical carcinoma |
| BLCA | Bladder Urothelial Carcinoma |
| BRCA | Breast invasive carcinoma |
| CESC | Cervical squamous cell carcinoma and endocervical adenocarcinoma |
| CHOL | Cholangiocarcinoma |
| COAD | Colon adenocarcinoma |
| DLBC | Lymphoid Neoplasm Diffuse Large B-cell Lymphoma |
| ESCA | Esophageal carcinoma |
| GBM | Glioblastoma multiforme |
| HNSC | Head and Neck squamous cell carcinoma |
| KICH | Kidney Chromophobe |
| KIRC | Kidney renal clear cell carcinoma |
| KIRP | Kidney renal papillary cell carcinoma |
| LAML | Acute Myeloid Leukemia |
| LGG | Brain Lower Grade Glioma |
| LIHC | Liver hepatocellular carcinoma |
| LUAD | Lung adenocarcinoma |
| LUSC | Lung squamous cell carcinoma |
| MESO | Mesothelioma |
| OV | Ovarian serous cystadenocarcinoma |
| PAAD | Pancreatic adenocarcinoma |
| PCPG | Pheochromocytoma and Paraganglioma |
| PRAD | Prostate adenocarcinoma |
| READ | Rectum adenocarcinoma |
| SARC | Sarcoma |
| SKCM | Skin Cutaneous Melanoma |
| STAD | Stomach adenocarcinoma |
| TGCT | Testicular Germ Cell Tumors |
| THCA | Thyroid carcinoma |
| THYM | Thymoma |
| UCEC | Uterine Corpus Endometrial Carcinoma |
| UCS | Uterine Carcinosarcoma |
| UVM | Uveal Melanoma |

**Table S2. The association between VCAN expression and clinicopathologic features in FUSCC cohort.**

| Characteristic | Low VCAN | High VCAN | p |
| --- | --- | --- | --- |
| All patients | 116 | 117 |  |
| Gender (%) |  |  | 1.000 |
| Female | 32 (13.7%) | 32 (13.7%) |  |
| Male | 84 (36.1%) | 85 (36.5%) |  |
| Age (%) |  |  | 0.046 |
| ＜ 65 year | 70 (30%) | 86 (36.9%) |  |
| ≥ 65 year | 46 (19.7%) | 31 (13.3%) |  |
| Tumor size (%) |  |  | 0.518 |
| ＜ 4cm | 67 (29.1%) | 74 (32.2%) |  |
| ≥ 4cm | 47 (20.4%) | 42 (18.3%) |  |
| TNM stage (%) |  |  | 0.341 |
| Ⅰ | 41 (17.6%) | 31 (13.3%) |  |
| Ⅱ | 28 (12%) | 33 (14.2%) |  |
| Ⅲ | 47 (20.2%) | 53 (22.7%) |  |
| Adjuvant chemotherapy (%) |  |  | 0.230 |
| No | 38 (16.3%) | 29 (12.4%) |  |
| Yes | 78 (33.5%) | 88 (37.8%) |  |
